# Dr. Frank J. Thornbury

**Published:** 1908-05

**Authors:** 


Dr. Frank J. Thornbury, formerly of Buffalo, died recently in a hospital at Rochester, N. Y., aged about fifty years. He graduated at the Medical College of Ohio, Medical Department University of Cincinnati, in 1889, and served for some years as assistant surgeon in the public health and marine hospital service.

